# Supplementary material for: Targeting Diet Quality at the Workplace: Influence on Cardiometabolic Risk
Source: Nutrients. 2021 Jun 30;13(7):2283. doi: 10.3390/nu13072283 (PMC8308315; doi:10.3390/nu13072283)
Supplement: Supplementary file 1 [file nutrients-13-02283-s001.zip › nutrients-1259079-supplementary.pdf]

Supplementary Table S1. Multiple linear regression models showing the independent associations of changes in diet quality index, physical activity level, exercise heart rate, waist circumference, medication use (lipids, hypertension, diabetes) and menopausal status on changes in lipid variables.

	Total R^2 x 100	Partial R^2 x 100 Δ DQ index	Partial R^2 x 100 Δ PAL	Partial R^2 x 100 Δ Exercise HR	Partial R^2 x 100 Δ Waist	Rx Lipids	Rx HTN	Rx Diabetes	Menopause
**Men**									
**Lipid profile (*n* = 678–694)**									
Δ Total cholesterol	10.3	1.4 ‡	1.0 ‡	–	7.3 *	–	0.5 ‡	–	NA
Δ LDL cholesterol	0.8	–	–	0.3	0.5	–	–	–	NA
Δ HDL cholesterol	1.8	0.4	–	1.5 ‡	–	–	–	–	NA
Δ Non-HDL cholesterol	13.1	0.5 ‡	1.3 ‡	–	10.5 *		0.8 ‡	–	NA
Δ Cholesterol/HDL cholesterol	10.4	–	1.0 ‡	0.5 ‡	8.4 *	–	0.5 ‡	–	NA
Δ Triglycerides	11.9	0.4	0.8 ‡	0.8 ‡	9.4 *	–	0.5	–	NA
**Women**									
**Lipid profile (*n* = 170–173)**									
Δ Total cholesterol	18.6	3.1 ‡	9.4 *	–	4.6 ‡	1.5	–	–	–
Δ LDL cholesterol	3.7	–	2.4 ‡	–	–	1.3	–	–	–
Δ HDL cholesterol	6.9	–	4.3 ‡	–	–	–	2.6 ‡	–	–
Δ Non-HDL cholesterol	17.2	3.0 ‡	8.3 †	–	4.8 ‡	1.1	–	–	–
Δ Cholesterol/HDL cholesterol	3.5	1.3	–	–	–	–	–	–	2.2 ‡
Δ Triglycerides	16.1	4.6 ‡	–	–	8.9 *	–	–	2.7 ‡	–

DQ: diet quality; HDL: high-density lipoprotein; HR: heart rate; LDL: low-density lipoprotein; NA: not applicable; PAL: physical activity level; *n*: range of participants; Rx: drug prescription; –: not included in the model due to lack of significance. ‡ $p \leq 0.05$; † $p < 0.001$; * $p < 0.0001$.
